# Hypothalamic Regulation of Cardiorespiratory Functions: Insights into the Dorsomedial and Perifornical Pathways

**DOI:** 10.3390/biology13110933

**Published:** 2024-11-15

**Authors:** Laura Carrillo-Franco, Marta González-García, Carmen Morales-Luque, Marc Stefan Dawid-Milner, Manuel Víctor López-González

**Affiliations:** 1 Department of Human Physiology, Faculty of Medicine, University of Málaga, 29010 Malaga, Spain; carmen6@uma.es (C.M.-L.); msdawid@uma.es (M.S.D.-M.); manuelvictor@uma.es (M.V.L.-G.); 2 Unit of Neurophysiology of the Autonomic Nervous System (CIMES), University of Málaga, 29010 Malaga, Spain; 3 Biomedical Research Institute of Málaga (IBIMA Plataforma BIONAND), 29010 Malaga, Spain

**Keywords:** autonomic nervous system, cardiorespiratory control, dorsomedial hypothalamic nucleus and perifornical area, stress, defense response

## Abstract

The **dorsomedial hypothalamus nucleus (DMH)** plays a pivotal role in the orchestration of sympathetic nervous system activities. Through its projections to the brainstem and pontomedullary nuclei, it controls heart rate, contractility, blood pressure, and respiratory activity, such as timing and volumes. The DMH integrates inputs from higher brain centers and processes these signals in order to modulate autonomic outflow accordingly. It has been demonstrated to be of particular significance in the context of stress responses, where it orchestrates the physiological adaptations that are necessary for all adaptative responses. The **perifornical region (PeF)**, which is closely associated with the DMH, also makes a contribution to autonomic regulation. The involvement of the PeF region in autonomic control is evidenced by its function in coordinating the autonomic and endocrine responses to stress, frequently in conjunction with the DMH. The DMH and the PeF do not function in an isolated manner; rather, they are components of a comprehensive hypothalamic network that integrates several autonomic responses. This neural network could serve as a target for developing therapeutic strategies in cardiovascular diseases.

## Simple Summary

The hypothalamus plays a critical role in the regulation of autonomic functions, acting as a central integrator of various physiological processes. Among its many regions, the dorsomedial hypothalamus and the perifornical region are particularly significant in the control of autonomic responses. This review highlights the critical roles of the dorsomedial hypothalamus and the perifornical region in autonomic control, emphasizing their importance in cardiorespiratory function.

## 1. Introduction

The stress response, triggered by aversive and threatening situations, induces immediate cardiovascular and respiratory changes, such as an increased blood pressure, heart rate, respiratory rate, and cutaneous vasoconstriction [1,2,3,4,5]. These autonomic responses enhance survival in threatening conditions [6]. This reflex, termed the “defense response”, is observed across various species, including humans and animal models [3,4,7].

The hypothalamus is a central structure in the defense response, facilitating rapid and adaptive responses. Early studies demonstrated that stimulation of specific posterior hypothalamic regions in cats triggers behavioral and physiological responses indicative of a defensive state [8]. Among the various hypothalamic nuclei, research has identified the dorsomedial hypothalamic nucleus (DMH) and the perifornical area (PeF) as key mediators of these autonomic responses. Together, these areas constitute the “Hypothalamic Defense Area” (HDA), as their stimulation elicits characteristic visceral and somatic changes [9].

These physiological strategies, which include autonomic, endocrine, and behavioral responses, have evolved over millions of years to be particularly effective in acute situations, typically characterized by fight-or-flight responses. However, it is critical to note that DMH/PeF-facilitated autonomic regulation is not limited to threat responses, but involves the autonomic adjustments of all adaptive responses, including motivational and emotional behaviors. Moreover, its involvement in these responses has become increasingly important, as the nature of psychological stressors has changed in today’s society, especially in terms of their severity and chronicity. This modern psychological stress has been strongly associated with an increased risk of cardiovascular problems, underscoring the urgent need to understand these evolving challenges [6,7,9].

Thus, the role of the DMH/PeF extends beyond immediate threat responses, encompassing broader physiological functions essential for adaptation to a variety of challenges. Specifically, extensive evidence has linked the DMH/PeF region to various autonomic functions, including the regulation of cardiovascular, respiratory, endocrine, and thermoregulatory responses and urinary bladder control [10,11,12,13,14]. In addition, the DMH/PeF also regulates sleep–wake cycles, circadian rhythms, homeostasis, reward-seeking behavior, and food intake [15,16,17].

These functions are executed through an extensive network of interconnections between the DMH/PeF and various cortical and brainstem regions. However, the precise nature of these connections is not yet fully understood. This study aims to review the input and output pathways that the DMH/PeF employs in regulating cardiorespiratory function in order to understand how these areas of the brain are involved in responses to threat situations and, above all, in a variety of adaptive and emotional behaviors. Considering the increasing number of cardiovascular problems and the apparent evidence that prolonged emotional stress may be responsible for these increases, clarifying the functional organization of this neural network could explain the neurophysiological bases of these alterations, potentially leading to new therapeutic strategies to address stress-related disorders and improve cardiovascular health.

## 2. Method

This narrative review applies a rigorous methodology to identify and synthesize key studies on the cardiorespiratory mechanisms regulated by the DMH/PeF. Given the exploratory and narrative nature of this review, systematic inclusion and exclusion criteria, as seen in systematic reviews, were not applied. Instead, studies were deliberately selected based on their scientific relevance, impact, and specific contributions to understanding DMH/PeF-related autonomic pathways. Below, we outline the study setting, the expertise of the research team, and the duration of this study, as well as the selection, evaluation, and quality control criteria applied to the reviewed literature.

### 2.1. Study Setting and Researchers’ Experience

This review was conducted within the Department of Human Physiology at the Faculty of Medicine, University of Málaga, a research environment with advanced facilities for physiological studies. The research team comprises specialists with extensive experience in autonomic nervous system studies, particularly in analyzing cardiorespiratory control mechanisms. This expertise enabled us to minimize biases and ensure reliability throughout the analysis.

### 2.2. Estimation of Bias and Quality of Outcome Measures

A comprehensive literature search was conducted using the PubMed database with keywords such as “autonomic nervous system”, “cardiorespiratory control”, “dorsomedial hypothalamic nucleus and perifornical area”, “psychological stress”, “defense response”, and “emotional stress”. The search targeted high-quality experimental and clinical studies published in peer-reviewed and high-impact journals. These studies address anatomical and functional aspects of cardiorespiratory control related to stress, with priority given to high-quality neuropharmacological, electrophysiological, neuroanatomical, and neuroimaging studies. For studies based on animal models, we prioritized those with findings that are interpretable and potentially translatable to human contexts, ensuring a thorough assessment of the physiological and neurobiological processes involved in cardiorespiratory regulation and stress responses.

In selecting articles, we carefully considered methodological rigor, result robustness, and the quality of outcome measures to ensure data validity. The inclusion criteria were as follows: (i) relevance to the topic, particularly studies exploring the relationship between the DMH/PeF and autonomic responses and cardiorespiratory changes associated with stress or threat situations; (ii) studies addressing DMH/PeF afferent and efferent pathways linked to cardiorespiratory control; and (iii) original studies or reviews with transparent and objective methodologies. Exclusion criteria included the following: (i) studies not directly related to the DMH/PeF or autonomic cardiorespiratory regulation; (ii) studies without relevant stress models (e.g., those not evaluating emotional or threat-based stress); (iii) studies with limited methodological robustness; (iv) studies lacking sufficient exploration of DMH/PeF pathways in the context of cardiorespiratory control; and (v) low-quality studies, such as those with small sample sizes or insufficient variable control. The “saturation” criterion determined the endpoint of the search, ending when no new articles presented novel findings or concepts.

Although this narrative review does not follow a formalized framework for assessing study bias or quality, we critically reviewed the studies to cover a broad spectrum of approaches and methods that contribute to the current understanding of DMH/PeF functions and its autonomic projections.

## 3. The Role of the DMH/PeF in Cardiorespiratory Function

The hypothalamus is a key regulator of stress-induced cardiorespiratory responses, a function supported by extensive research. Early studies demonstrated that electrical stimulation of the hypothalamus leads to notable increases in sympathetic activity, heart rate, blood pressure, and respiratory activity, identifying it as a central structure in the autonomic response to stress.

Among the hypothalamic regions, the DMH/PeF has been recognized as the primary regulator of these autonomic changes. Electrical stimulation of the DMH/PeF evokes profound cardiorespiratory alterations, making this region a central focus for understanding the body’s physiological reaction to stress. Notably, these changes are not limited to electrical stimulation; chemical stimulation, such as the disinhibition of the DMH/PeF with the GABAA receptor antagonist bicuculline, also results in increased heart rate and blood pressure, which are proportional to the doses administered [9,18,19].

In contrast, the inhibition of DMH/PeF activity leads to significant reductions in key autonomic parameters. Studies in conscious rats have shown that DMH/PeF blockade results in decreased pressor responses, tachycardia, skeletal muscle blood flow, and renal sympathetic activity [20,21]. Furthermore, the suppression of respiratory responses to excitatory and stressful stimuli during DMH/PeF inhibition highlights the critical role of this region in modulating respiratory rates under stress [2].

Interestingly, the cardiorespiratory changes elicited by DMH/PeF stimulation in anesthetized rats closely resemble those observed during natural stress responses, reinforcing the idea that this region is integral to autonomic regulation [9]. Additionally, acute stress has been linked to increased c-Fos expression in the DMH, indicating the activation of this area during stressful conditions [22].

Beyond fast autonomic responses, the DMH/PeF also plays a crucial role in the re-adjustment of reflexes that regulate blood pressure. For example, during stress, it facilitates the adjustment of the baroreceptor reflex to accommodate elevated blood pressure levels, a necessary adaptation for the body’s defense response [23]. Additionally, the DMH/PeF is involved in enhancing the chemoreflex [24,25], further demonstrating its multifaceted role in cardiorespiratory control.

In summary, the DMH/PeF emerges as a central hub in orchestrating the body’s cardiorespiratory responses to stress. By influencing heart rate, blood pressure, and respiratory activity, and through its interactions with other autonomic reflexes, this hypothalamic region ensures a coordinated physiological response to environmental challenges.

### 3.1. Afferent Pathways to the DMH-PeF: Essential Connections for Cardiorespiratory Control

**Figure 1:**
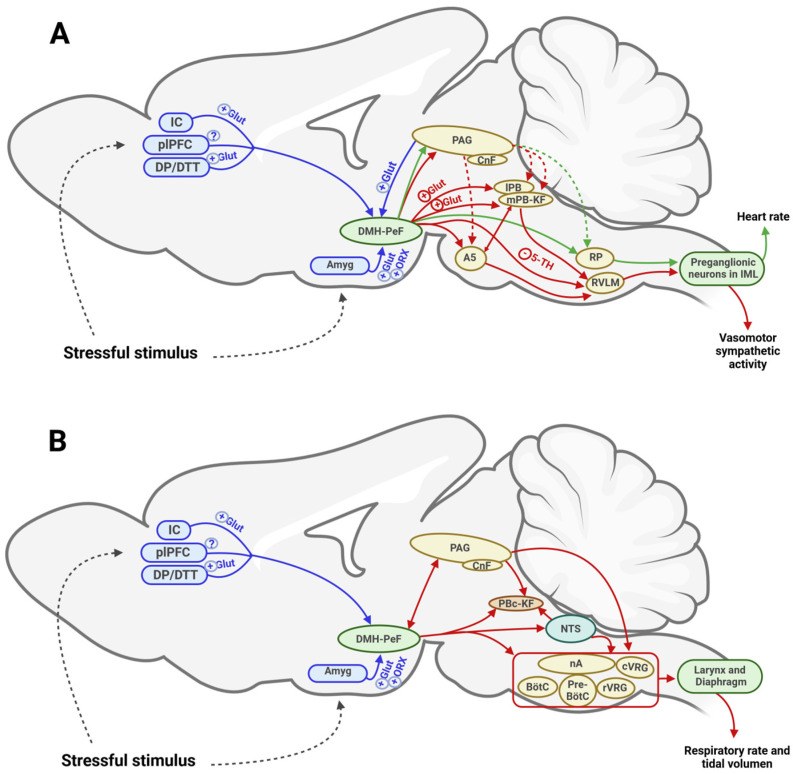
**Schematic representation of the primary cortical, mesencephalic, and pontomedullary regions involved in the cardiorespiratory response mediated by the dorsomedial hypothalamic nucleus and perifornical area (DMH/PeF).** (**A**) **Cardiovascular function.** The figure illustrates the input pathways (blue arrows) and the output pathways involved in vasomotor sympathetic activity (red arrows) and heart rate (green arrows) regulated by DMH/PeF, as well as the neurotransmitter involved in those connections. The pressor response to HDM/PeF activation may be mediated by direct downstream projections to preganglionic neurons in the RVLM and by activation of ionotropic glutamate receptors located in the lPB/mPB-KF and A5 (red solid arrows). At the same time, indirect connections to the lPB/mPB-KF and A5 via the PAG may be involved (red dashed arrows). The cardiac component is regulated by a direct downstream pathway from the HDM/PeF to the RP (green solid arrows). An alternative indirect pathway via the PAG is thought to be involved (green dashed arrows). (**B**) Respiratory function. This diagram illustrates the possible interactions between the DMH/PeF and PAG, both with each other and with medullary–pontine respiratory nuclei, in relation to changes in respiratory rate. 5-HT: 5-hydroxytryptamine; A5: region of the A5 noradrenergic cells; Amyg: amygdala; BötC: Bötzinger complex; CnF: cuneiform nucleus; cVRG: caudal ventral respiratory group; DMH/PeF: dorsomedial hypothalamus and perifornical area; DP/DTT: dorsal peduncular cortex and dorsal tenia tecta in the ventromedial prefrontal cortex; Glut: glutamate; IC: insular cortex; IML: intermediolateral cell column; lPB: lateral parabrachial nucleus; mPB-KF: medial parabrachial nucleus and Kölliker-Fuse nucleus; mPFC: medial prefrontal cortex; mPOA: medial pre-optic area; nA: nucleus ambiguus; NTS: nucleus of tractus solitarius; ORX: orexin; PAG: periaqueductal gray matter; PBc: parabrachial complex; pre-BötC: pre-Bötzinger complex; RP: raphe pallidus; RVLM: rostroventrolateral medulla; rVRG: rostral ventral respiratory group; (+): excitatory neurotransmitter; (-) inhibitory neurotransmitter; (?) unknown neurotransmitter. *Created in BioRender. Carrillo Franco, L. (2024) BioRender.com/k38n231*.

The DMH/PeF modulates cardiorespiratory activity in response to stress by integrating information from multiple brain structures associated with autonomic functions. The key input pathways to the DMH/PeF are detailed below, organized by their anatomical locations: cortical regions, the amygdala and other limbic areas, the periaqueductal gray matter (PAG) in the midbrain, and the nucleus of tractus solitarius (NTS) within the medulla oblongata (see Figure 1).

#### 3.1.1. Cortical Regions

The insular cortex (IC) has been identified as a major input to the posterior DMH/PeF. Recent findings indicate that the intermediate region of the posterior IC can influence DMH/PeF-mediated sympathetic activity through direct glutamatergic projections, specifically via *N*-methyl-d-aspartate receptors (NMDARs) [26]. Evidence suggests a functional specialization within the IC, where the right hemisphere is involved in cardiac sympathetic regulation and the left hemisphere modulates cardiac vagal activity [27]. Studies in rodents and humans support the role of the IC in autonomic cardiovascular control, with damage to this region associated with cardiac dysfunction [28,29,30,31,32].

Beyond the insular cortex, the prelimbic prefrontal cortex (plPFC) directly connects to the DMH/PeF and significantly influences respiratory responses to stress. Inhibition of the plPFC has been shown to reduce panic behaviors triggered by DMH/PeF activation [2]. Additionally, the dorsal peduncular cortex and dorsal tenia tecta (DP/DTT) have been proposed to activate a glutamatergic pathway directed towards the DMH during stressful stimuli, highlighting a potential cortical influence on autonomic responses [33].

#### 3.1.2. Amygdala and Limbic Structures

The amygdala, a key limbic structure associated with emotional processing and autonomic responses, is another important input to the DMH/PeF [34]. Blocking glutamate receptors in the DMH/PeF has been shown to suppress cardiovascular responses induced by chemical stimulation of the amygdala [35]. Additionally, the amygdala provides orexinergic input to the DMH/PeF, further influencing cardiorespiratory activity [36]. Recent neuroimaging studies have reinforced the amygdala’s role in modulating sympathetic responses to stress, indicating that its projections to the DMH adjust cardiovascular behaviors in response to perceived threats [37,38].

#### 3.1.3. Periaqueductal Gray Matter (PAG)

The PAG, a mesencephalic region known for its role in integrating defensive behaviors and autonomic responses to threats, has direct projections to the DMH [39]. These projections are believed to be glutamatergic, modulating cardiovascular responses mediated by the DMH/PeF. Notably, activity in the lateral and dorsolateral columns of the PAG (l/dlPAG) can trigger physiological responses that depend on DMH neuronal activity [40]. Studies have shown that the microinjection of muscimol and NBQX-Ap5 into the DMH/PeF reduces heart rate, blood pressure, and body temperature increases induced by l/dlPAG stimulation [41]. These findings underscore the PAG-DMH/PeF pathway’s role in mediating stress responses.

#### 3.1.4. Nucleus of Tractus Solitarius (NTS)

The NTS appears to play an essential role as an afferent to the DMH/PeF during stress, facilitating increases in respiratory rate and baroreflex adjustment [24,42,43,44,45]. The specific mechanisms by which the NTS interacts with the DMH/PeF to mediate cardiorespiratory control are elaborated in the following sections.

In summary, the DMH/PeF integrates input from multiple brain regions, each contributing to its role in modulating autonomic responses to stress. Understanding these connections is crucial for comprehending the mechanisms underlying cardiorespiratory regulation during periods of heightened arousal.

### 3.2. Efferent Pathways from the Dorsomedial and Perifornical Hypothalamus: A Unified Framework for Cardiorespiratory Regulation

**Figure 2:**
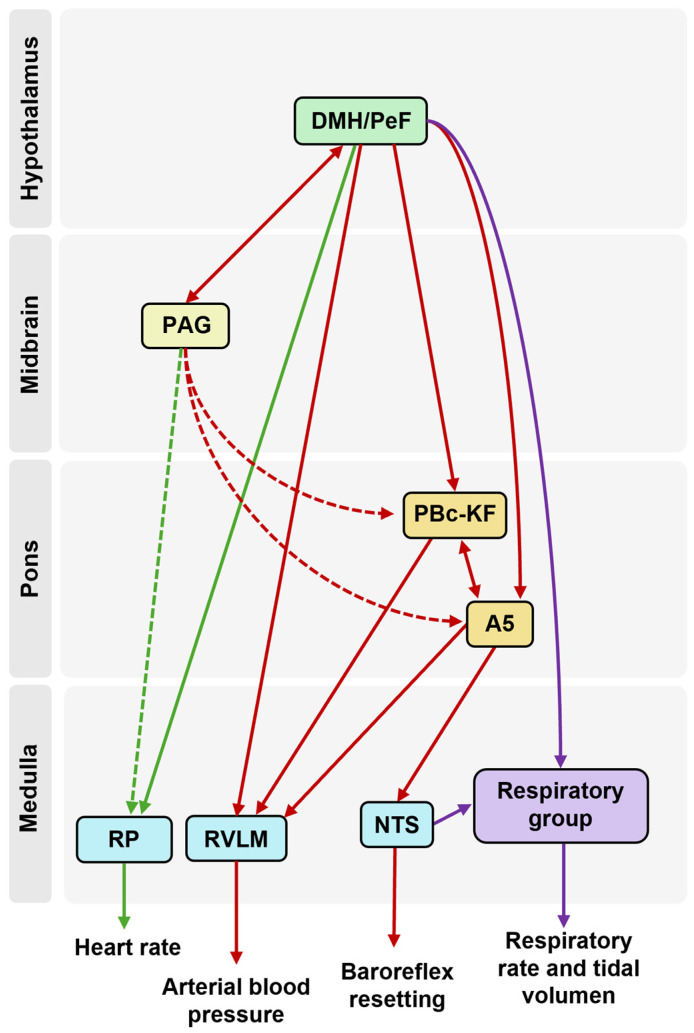
**Simplified diagram of the neural mechanisms in cardiovascular and respiratory control regulated by DHM/PeF.** This diagram highlights pathways involved in the regulation of tidal volume and respiratory rate (purple arrows), heart rate (green arrows), and blood pressure and baroreceptor reflex adjustment (red arrows). Dashed arrows show indirect connections.

Anatomical tracing studies indicate that the DMH/PeF region does not directly connect to preganglionic sympathetic neurons in the spinal cord [46,47]. Thus, its influence on cardiorespiratory activity is mediated through ascending and descending sympathoexcitatory pathways that interact with premotor neurons. The potential pathways through which these autonomic responses are modulated are outlined below, categorized by their anatomical locations: mesencephalic structures, pontine nuclei, and medullary nuclei (see Figure 1 and Figure 2).

#### 3.2.1. Mesencephalic Structures

The PAG is a critical mesencephalic region that integrates defensive behaviors and autonomic responses to threats. It receives input from cortical areas, including the prefrontal cortex and the amygdala, and sends outputs to cardiorespiratory pontomedullary nuclei [48]. The PAG is composed of four columns—lateral (lPAG), dorsolateral (dlPAG), dorsomedial, and ventrolateral (vlPAG)—each contributing to different aspects of autonomic regulation [49,50].

Among these, the l/dlPAG serves as an important relay for DMH/PeF-induced cardiovascular responses. The DMH/PeF projects to the l/dlPAG, which integrates these signals and redistributes the vasomotor component to the rostral ventrolateral medulla (RVLM) and the cardiac component to the raphe pallidus (RP) [51,52]. Although the l/dlPAG lacks direct connections to the intermediolateral cell column (IML) of the spinal cord, it is likely involved in modulating cardiovascular responses through indirect pathways [51].

Experimental evidence supports the role of the l/dlPAG in mediating DMH/PeF-induced changes in autonomic function. Microinjection studies have shown that administering muscimol (a GABA agonist) and NBQX-Ap5 (an ionotropic glutamate receptor antagonist) into the DMH/PeF significantly reduces increases in heart rate, blood pressure, and body temperature typically induced by excitatory amino acid stimulation of the l/dlPAG [41]. Similarly, DMH/PeF inhibition suppresses cardiovascular and respiratory responses triggered by PAG activation [40]. This suggests a dynamic interplay between the DMH/PeF and the l/dlPAG, where the latter serves as a mediator that relays and refines autonomic signals to other brainstem cardiorespiratory centers. Morphofunctional studies further substantiate this role, showing direct neural connections from the l/dlPAG to the DMH, reinforcing the idea that the l/dlPAG integrates signals for proper autonomic response [53,54].

The l/dlPAG also contains 5-HT1A receptors, which are known to modulate autonomic responses. Activation of these receptors has been shown to decrease tachycardia and hypertension triggered by DMH/PeF stimulation [55]. It is proposed that the serotonergic component of the l/dlPAG originates in the vlPAG, a subregion recognized for its role in inhibiting the defense response [56]. This implies that 5-HT1A receptors in the l/dlPAG may reduce the transmission of signals to sympathetic premotor neurons, thereby modulating the descending cardiovascular pathways from the DMH/PeF [18,19,57].

Beyond cardiovascular regulation, the l/dlPAG is also critical for respiratory control, as demonstrated by the induction of tachypnea upon its activation [58]. This respiratory modulation is mediated via projections from the l/dlPAG to pontomedullary respiratory nuclei, specifically the Kölliker-Fuse (KF) nucleus and the nucleus retroambiguus (nRA). The l/dlPAG’s influence on the KF nucleus is particularly important, as the KF nucleus adjusts the transition between inspiration and expiration, thereby fine-tuning the respiratory pattern in response to autonomic demands [59]. By acting on neurons that modulate abdominal pressure, the l/dlPAG facilitates the switch from passive to active breathing, an adjustment crucial during stress-induced states [60,61].

The functional connections between the DMH/PeF, l/dlPAG, and the KF nucleus highlight the complex yet coordinated nature of cardiorespiratory regulation during stress. This interplay ensures that autonomic responses, such as changes in heart rate, blood pressure, and respiratory rhythm, are finely balanced, allowing the organism to adapt effectively to varying environmental challenges.

#### 3.2.2. Pontine Nuclei

The pons contains key nuclei involved in DMH/PeF-mediated regulation of cardiorespiratory activity, primarily the parabrachial complex (PBc) and the region of the A5 noradrenergic cells (A5). These pontine nuclei play pivotal roles in modulating both respiratory and cardiovascular responses, serving as crucial relay points for signals descending from the DMH/PeF [24].

The PBc itself consists of several subdivisions, including the lateral parabrachial nucleus (lPB), the medial parabrachial nucleus (mPB), and the KF nucleus [62]. Each subdivision has a specific function in the control of respiratory patterns and cardiovascular activity. The lPB has been identified as particularly influential in driving tachypnea. Activation of glutamatergic neurons within the lPB leads to an increased respiratory rate [63]. This function is essential during the defense response, as the body demands rapid adaptations to ensure an adequate oxygen supply for the heightened metabolic activity associated with stress.

Conversely, the mPB and KF nucleus contribute to the modulation of breathing by inducing bradypnea when activated [63]. The KF nucleus plays a critical role in coordinating the transition between inspiration and expiration, thereby fine-tuning the respiratory rhythm in response to autonomic demands. By acting on neurons responsible for respiratory pattern generation, the PBc-KF complex helps shift from passive to active breathing, a necessary adaptation during stress-induced states. Both nuclei, which form the pontine respiratory group (PRG), perform this function through reciprocal connections with the dorsal respiratory group (DRG) and ventral respiratory group (VRG) [64,65]. Furthermore, research has shown that both lPB and KF nuclei receive direct projections from the DMH/PeF, emphasizing their importance as mediators of DMH/PeF-driven respiratory changes [66,67].

The functional distinction between these parabrachial subdivisions has been confirmed through neuropharmacological studies. For example, using muscimol to inhibit lPB neurons suppresses the tachypnea induced by DMH/PeF activation, indicating that the lPB is directly involved in mediating the respiratory effects of DMH/PeF activity [67]. Similarly, activation of the lPB has been found to exert an efficient control over cardiovascular responses by influencing neurons in the RVLM that project to the sympathetic nervous system. This indicates that the lPB serves as a key relay point, translating DMH/PeF signals into both respiratory and cardiovascular adjustments. Importantly, the involvement of glutamate, acting on ionotropic receptors within the PBc, underscores its role in mediating the effects of DMH/PeF, contributing to the rapid autonomic adjustments during stress [63,67,68].

The A5, containing both catecholaminergic and non-catecholaminergic neurons, adds another layer of complexity to this network. Its unique composition allows it to regulate cardiovascular and respiratory functions separately [69]. The A5 is known to receive input from the DMH/PeF and has been shown to interact closely with the PBc. Inhibition of the A5 has been demonstrated to significantly attenuate the cardiorespiratory responses induced by PBc activation, highlighting the interplay between these pontine nuclei in autonomic regulation [63,70,71]. This interaction suggests that the A5 acts as a modulatory hub that refines the signals received from the DMH/PeF, contributing to the precise control of cardiovascular responses, including blood pressure regulation.

Additionally, the A5 region’s catecholaminergic neurons influence sympathetic outflow, facilitating blood pressure adjustments during stress [72]. The A5 has connections with the RVLM and plays a part in the descending autonomic pathways that originate in the DMH/PeF. By mediating the communication between the DMH/PeF and the RVLM, the A5 helps modulate sympathetic tone, ensuring a coordinated cardiovascular response [70,71].

The PBc is also actively involved in facilitating the chemoreflex, which is crucial for maintaining homeostasis during stress. The DMH/PeF has been shown to enhance chemoreceptor reflexes by exciting neurons in the NTS. The NTS then sends projections to the PBc, where the lPB, mPB, and KF nuclei interact to modify respiratory rates. Importantly, the lPB appears to exert a more dominant effect on the chemoreceptor reflex, modulating both the respiratory and cardiovascular aspects of the response. When the body faces situations that require an increase in respiratory rate, the DMH/PeF-PBc pathway becomes activated, facilitating the adjustment of breathing patterns in alignment with the autonomic state [42,43,44,45].

To summarize, the PBc-KF complex and the A5 work in concert with the DMH/PeF to integrate cardiorespiratory signals. The lPB is particularly involved in inducing tachypnea and modulating cardiovascular responses, while the mPB and KF nucleus focus on fine-tuning respiratory patterns. The A5 serves as a modulatory node that refines the signals descending from the DMH/PeF, contributing to the maintenance of blood pressure and the facilitation of autonomic adjustments during stress. Together, these pontine nuclei ensure that cardiorespiratory responses are appropriately synchronized, allowing the organism to adapt to varying environmental and physiological challenges effectively.

#### 3.2.3. Medullary Nuclei

The medulla oblongata is a critical structure in cardiorespiratory regulation, acting as a major relay station for autonomic signals descending from the DMH/PeF. The main medullary nuclei involved in this process include the RVLM, the RP, and the NTS. These nuclei house sympathetic premotor neurons responsible for modulating vasomotor tone, heart rate, and respiratory activity, thereby playing a crucial role in generating adaptive autonomic responses to stress.

The RVLM is pivotal in controlling sympathetic vasomotor activity, thereby directly influencing blood pressure regulation. It contains sympathetic premotor neurons that project to the IML of the spinal cord, where they modulate the activity of preganglionic sympathetic neurons [73,74]. Stimulation of the DMH/PeF leads to an increase in the firing rate of RVLM neurons, resulting in enhanced sympathetic outflow and peripheral vasoconstriction, culminating in elevated blood pressure [19]. This mechanism is crucial for the defense response, where rapid redistribution of blood flow to essential organs, such as the brain and skeletal muscles, is necessary.

The influence of the DMH/PeF over the RVLM is mediated through glutamatergic pathways. Activation of the DMH/PeF stimulates RVLM neurons via the release of excitatory amino acids, facilitating sympathetic tone and thereby contributing to the autonomic changes seen during stress [57]. Additionally, there is evidence suggesting a bidirectional relationship between these structures. Studies have shown that microinjection of GABAergic agents into the RVLM can inhibit the pressor response evoked by DMH/PeF stimulation, indicating that the RVLM not only acts as an intermediary but also modulates the autonomic tone in response to DMH/PeF activity. Although the DMH/PeF is known to project to the RVLM, evidence indicates that the PAG also sends projections to the RVLM [75]. Therefore, the DMH/PeF may indirectly modulate arterial pressure and sympathetic activity in the RVLM via the PAG.

Moreover, the orexin system within the DMH/PeF significantly impacts the RVLM’s activity. Orexin, a neuropeptide produced in the lateral hypothalamic area, including the DMH/PeF, plays a role in enhancing sympathetic drive during stress. Microinjection of orexin A and B into the RVLM produces sympathoexcitatory cardiovascular effects, suggesting that orexin could potentiate the cardiovascular responses to stress [76]. This effect appears to be primarily mediated by ORX2 receptors, although ORX1 receptors also contribute to the response [77]. The presence of orexinergic projections underscores the complexity of DMH/PeF-RVLM interactions in autonomic regulation.

The RP plays an equally critical role, particularly in the modulation of heart rate. It contains a population of serotonergic neurons that project to cardiac preganglionic neurons in the spinal cord, influencing sympathetic cardiac output. Activation of the DMH/PeF leads to excitation of RP neurons, resulting in tachycardia, which is a hallmark of the defense response [19,57,78]. Anatomical studies have identified that most projections to the RP originate from the dorsal region of the DMH/PeF [79], highlighting a specialized pathway for cardiac modulation. While an indirect pathway through the PAG has been reported [51,52], other authors propose that tachycardia induced by emotional stress relies on the DMH/PeF-RP pathway [78].

The NTS is the primary integrative center for visceral sensory information, including input from baroreceptors, chemoreceptors, and pulmonary stretch receptors. The NTS receives direct projections from the DMH/PeF and plays a central role in the adjustment of the baroreceptor reflex during stress. This reflex adjustment is essential for accommodating the elevated blood pressure levels typically encountered during the defense response, ensuring effective cardiovascular control under heightened physiological demands [80,81].

The DMH/PeF modulates the activity of the NTS through both direct and indirect pathways. Directly, the DMH/PeF inhibits NTS neurons via activation of GABAA receptors. When the DMH/PeF is activated, it releases glutamate onto ionotropic glutamatergic receptors located on GABAergic interneurons within the NTS. This prompts the interneurons to release GABA, which in turn inhibits the cholinergic vagal preganglionic neurons in the dorsal motor nucleus of the vagus. This mechanism reduces parasympathetic activity, blunting the baroreceptor reflex and allowing a more flexible range of blood pressure regulation during stress [43,44].

Moreover, leptin, an adipocyte-derived hormone, has been found to influence respiratory activity through its actions on the DMH/PeF. Specific neurons within the DMH/PeF-expressing LEPRb+ receptors project to the dorsal raphe, where they modulate respiratory rate [82]. This pathway affects leptin-induced facilitation of the chemoreflex and the activation of motor neurons controlling tongue muscles, which aid in maintaining an open airway during breathing [83]. The interaction between leptin and the DMH/PeF underscores the nucleus’s multifaceted role in autonomic and respiratory regulation.

The DRG, located in the NTS and VRG, including the nRA and the pre-Bötzinger complex, are involved in the respiratory adjustments mediated by the DMH/PeF. In response to stress, the DMH/PeF facilitates the chemoreflex by exciting NTS neurons, which then project to these respiratory centers. This results in an increased respiratory rate and tidal volume, optimizing the physiological state to meet the metabolic demands of the defense response [59].

The DMH/PeF’s modulation of the NTS and its downstream connections to the DRG and VRG ensures that cardiorespiratory responses are dynamically adjusted to suit the organism’s needs during stress. This dynamic adjustment involves both direct modulation of the NTS and indirect pathways, including connections with the dorsolateral PAG and the cuneiform nucleus, which further enhance the medullary network’s regulatory capacity [48,84].

In summary, the medullary nuclei—RVLM, RP, and NTS—along with the dorsal and ventral respiratory groups constitute an integrated network through which the DMH/PeF exerts its control over cardiorespiratory functions. By modulating the RVLM and RP, the DMH/PeF influences vasomotor tone and heart rate, while interactions with the NTS, DRG, and VRG adjust respiratory patterns and reflexes. This coordinated action enables the organism to respond effectively to stress, highlighting the medulla’s central role in autonomic regulation.

## 4. Clinical Implications

The high prevalence of individuals with cardiovascular diseases, such as hypertension and cardiac arrhythmias, is a well-documented problem. Although these disorders are attributable to genetic factors and other risks, there is evidence suggesting that daily emotional and psychosocial stress may contribute to the exacerbation of these conditions [85,86].

The increase in anxiety disorders has been specifically associated with hypertension, indicating a direct link between emotional state and cardiovascular health. This relationship is due to the typical sympathetic activation seen in anxiety, which includes increased cardiac output and peripheral resistance [87,88]. These reactions are similar to those observed in humans during stress or threat situations, which provoke increased secretion of ACTH and respiratory and cardiovascular changes, including hypertension, tachycardia, and elevated cardiac output [89].

All of these health issues have been associated with dysfunction of the DMH/PeF. A study suggests that sustained stress may trigger an increase in both sympathetic activity and long-term blood pressure, indicating that the DMH/PeF may influence these cardiovascular alterations [90]. Although it remains unclear whether the dysfunction originates in the DMH/PeF itself or in its afferent/efferent pathways, some authors propose that serotonin 5-HT1A receptors may be involved in this process. It has been demonstrated that sympathetic excitation and tachycardia induced by the DMH/PeF can be inhibited through the activation of these receptors, highlighting their importance in the regulation of the stress response [91].

Furthermore, these authors found that prolonged exposure to a stressor induces cardiovascular changes similar to those triggered by anxiety. They also suggest that chronic activation of 5-HT3 receptors in the context of stress may further exacerbate these responses. This underscores the necessity to consider the management of emotional and psychosocial stress as a preventive and therapeutic approach in the care of patients with cardiovascular diseases [91].

## 5. Limitations

Given the narrative nature of this review, there are inherent limitations in scope and methodology. First, although a careful selection of high-quality and scientifically relevant studies was conducted, we did not apply systematic inclusion or exclusion criteria, which may introduce some selection bias. Unlike systematic reviews, no formal assessment of study quality or potential biases in the reviewed methodologies was conducted. This is particularly relevant for the substantial proportion of studies derived from animal models, which, while providing valuable mechanistic insights, may not fully generalize to clinical applications in humans [3,35].

Another consideration is the variability in methodological approaches used in the reviewed studies. The included works range from anatomical tracing studies [47] to pharmacological investigations [41,51,70], which, while enriching the review, may introduce differences in result interpretation due to the heterogeneity of techniques applied.

This review also covers a broad spectrum of topics related to cardiorespiratory regulation, from the function of orexin in the DMH/PeF [77] to leptin-mediated modulation of respiratory activity [83]. However, specific areas, such as the detailed neurochemical interactions between neurotransmitters and receptors within the DMH/PeF and functional lateralization observed in certain studies [7], still require further investigation for a more comprehensive understanding. These topics represent critical areas for future studies to unravel the complexity of the DMH/PeF in autonomic regulation.

A limitation identified in this review is the limited number of studies examining the relationship between DMH/PeF activity and its impact on human stress-associated pathologies, such as hypertension and anxiety [86,87]. Additionally, the scarcity of longitudinal data on the effects of chronic stress on autonomic function restricts our understanding of how these findings might be applied in clinical contexts.

## 6. Conclusions and Future Directions

The DMH-PeF serves as a central hub in the orchestration of cardiorespiratory responses, integrating signals from multiple brain regions to facilitate rapid and adaptive changes during stress. This review highlights the complexity and interconnectivity of the neural circuits that modulate autonomic functions, emphasizing the DMH/PeF’s multifaceted role in regulating cardiovascular and respiratory activities.

The evidence presented shows that the DMH/PeF exerts its influence on cardiorespiratory function through an extensive network of interconnections with various brainstem structures. The RVLM and RP are key targets for direct or indirect modulation by the DMH/PeF, ensuring the proper adjustment of blood pressure and heart rate during stressful conditions. Furthermore, the PBc, including the KF nucleus, and the A5 are integral in translating DMH/PeF signals into respiratory responses, such as tachypnea, which is crucial for managing metabolic demands.

The role of orexin and leptin in the DMH/PeF further illustrates the versatility of this hypothalamic region in coordinating autonomic outputs. Orexinergic projections modulate cardiovascular responses through their action on the RVLM and RP, suggesting a direct link between hypothalamic activity and sympathetic tone enhancement during stress. Similarly, the leptin-induced modulation of respiratory activity through the DMH/PeF points to a complex integration of metabolic signals within the autonomic response network. These neurochemical mechanisms underline the DMH/PeF’s involvement in both homeostatic and adaptive processes, bridging metabolic, cardiovascular, and respiratory regulation.

Additionally, the DMH/PeF’s interaction with the NTS plays a pivotal role in the readjustment of the baroreceptor and chemoreceptor reflexes. This modulation ensures flexibility in autonomic control, allowing the organism to accommodate the increased blood pressure and altered respiratory patterns characteristic of stressful states. The connections with dorsal and ventral respiratory groups further demonstrate the DMH/PeF’s central role in respiratory control, adapting breathing patterns to meet physiological demands.

Despite the significant progress in understanding the DMH/PeF’s role in autonomic regulation, several aspects remain to be fully elucidated. Future research could benefit from the following:

- **Mapping Specific Neural Pathways**: A more detailed characterization of the specific neural pathways, including the precise neurotransmitters and receptors involved in DMH/PeF-driven responses, would help clarify how the DMH/PeF integrates information from various brain regions.
- **Exploring Neurochemical Interactions**: Investigating the interactions between orexin, leptin, and other neuropeptides within the DMH/PeF could provide insights into how metabolic states influence autonomic regulation during stress.
- **Examining Functional Asymmetry**: Given evidence suggesting lateralization in the DMH/PeF’s influence on cardiovascular control, understanding the functional implications of this asymmetry could enhance our knowledge of individualized autonomic responses to stress.
- **Integrating Metabolic and Autonomic Studies**: Exploring how the DMH/PeF integrates signals related to metabolic status (e.g., leptin levels) with autonomic outputs might reveal novel mechanisms by which the body maintains homeostasis in response to changing internal and external environments.

Furthermore, evidence suggests that chronic emotional and psychosocial stress may significantly contribute to the onset and progression of cardiovascular conditions. A deeper understanding of the role of the DMH/PeF in autonomic regulation could position these regions as potential therapeutic targets for treating stress-related disorders such as anxiety and hypertension.

## Data Availability

No new data were created or analyzed in this study. Data sharing is not applicable to this article.

## Abbreviations

A5	Region of the A5 noradrenergic cells
dl PAG	Dorsolateral Periaqueductal Gray Matter
DMH/PeF	Dorsomedial Hypothalamic Nucleus and Perifornical Area
DRG	Dorsal Respiratory Group
HDA	Hypothalamic Defense Area
IC	Insular Cortex
IML	Intermediolateral Cell Column
KF	Kölliker-Fuse Nucleus
l/dlPAG	Lateral/Dorsolateral Periaqueductal Gray Matter
lPB	Lateral Parabrachial Nucleus
mPB	Medial Parabrachial Nucleus
nRA	Nucleus Retroambiguus
NTS	Nucleus of Tractus Solitarius
PAG	Periaqueductal Gray Matter
PBc	Parabrachial Complex
Pl PFC	Prelimbic Prefrontal Cortex
PRG	Pontine Respiratory Group
RP	Nucleus Raphe Pallidus
RVLM	Rostral Ventrolateral Medulla
VRG	Ventral Respiratory Group

## References

[1] Nakamura K., Morrison S.F. (2022). Central sympathetic network for thermoregulatory responses to psychological stress. Auton. Neurosci..

[2] Bondarenko E., Beig M.I., Hodgson D.M., Braga V.A., Nalivaiko E. (2015). Blockade of the dorsomedial hypothalamus and the perifornical area inhibits respiratory responses to arousing and stressful stimuli. Am. J. Physiol. Regul. Integr. Comp. Physiol..

[3] Bondarenko E., Hodgson D.M., Nalivaiko E. (2014). Amygdala mediates respiratory responses to sudden arousing stimuli and to restraint stress in rats. Am. J. Physiol. Regul. Integr. Comp. Physiol..

[4] Mohammed M., Kulasekara K., De Menezes R.C., Ootsuka Y., Blessing W.W. (2013). Inactivation of neuronal function in the amygdaloid region reduces tail artery blood flow alerting responses in conscious rats. Neuroscience.

[5] Kabir M.M., Beig M.I., Baumert M., Trombini M., Mastorci F., Sgoifo A., Walker F.R., Day T.A., Nalivaiko E. (2010). Respiratory pattern in awake rats: Effects of motor activity and of alerting stimuli. Physiol. Behav..

[6] Fontes M.A.P., Xavier C.H., de Menezes R.C.A., DiMicco J.A. (2011). The dorsomedial hypothalamus and the central pathways involved in the cardiovascular response to emotional stress. Neuroscience.

[7] Fontes M.A.P., Filho M.L., Santos Machado N.L., de Paula C.A., Souza Cordeiro L.M., Xavier C.H., Ribeiro Marins F., Herdenson L., Macefield V.G. (2017). Asymmetric sympathetic output: The dorsomedial hypothalamus as a potential link between emotional stress and cardiac arrhythmias. Auton. Neurosci..

[8] Hess W.R., Brugger M. (1943). Das subkortikale Zentrum der affektiven Abwehrreaktion. Helv. Physiol. Acta.

[9] DiMicco J.A., Samuels B.C., Zaretskaia M.V., Zaretsky D.V. (2002). The dorsomedial hypothalamus and the response to stress: Part renaissance, part revolution. Pharmacol. Biochem. Behav..

[10] Dampney R.A. (2016). Central neural control of the cardiovascular system: Current perspectives. Adv. Physiol. Educ..

[11] Fukushi I., Yokota S., Okada Y. (2018). The role of the hypothalamus in modulation of respiration. Respir. Physiol. Neurobiol..

[12] Pang Z.P., Han W. (2012). Regulation of synaptic functions in central nervous system by endocrine hormones and the maintenance of energy homoeostasis. Biosci. Rep..

[13] Nakamura K. (2011). Central circuitries for body temperature regulation and fever. Am. J. Physiol. Regul. Integr. Comp. Physiol..

[14] Rocha I., Silva-Carvalho L., Spyer K.M. (2004). Effect of stimulation of anterior hypothalamic area on urinary bladder function of the anesthetized rat. Clin. Auton. Res..

[15] Li L., Zhang M.Q., Sun X., Liu W.-Y., Huang Z.-L., Wang Y.-Q. (2022). Role of Dorsomedial Hypothalamus GABAergic Neurons in Sleep-Wake States in Response to Changes in Ambient Temperature in Mice. Int. J. Mol. Sci..

[16] Ramirez-Plascencia O.D., De Luca R., Machado N.L.S., Eghlidi D., Khanday M.A., Bandaru S.S., Raffin F., Vujovic N., Arrigoni E., Saper C.B. (2024). A hypothalamic circuit for circadian regulation of corticosterone secretion. Res. Sq..

[17] Houtz J., Liao G.Y., An J.J., Xu B. (2021). Discrete TrkB-expressing neurons of the dorsomedial hypothalamus regulate feeding and thermogenesis. Proc. Natl. Acad. Sci. USA.

[18] Fontes M.A.P., Tagawa T., Polson J.W., Cavanagh S.J., Dampney R.A.L. (2001). Descending pathways mediating cardiovascular response from dorsomedial hypothalamic nucleus. Am. J. Physiol. Heart Circ. Physiol..

[19] Horiuchi J., McAllen R.M., Allen A.M., Killinger S., Fontes M.A.P., Dampney R.A.L. (2004). Descending vasomotor pathways from the dorsomedial hypothalamic nucleus: Role of medullary raphe and RVLM. Am. J. Physiol. Regul. Integr. Comp. Physiol..

[20] Queiroz E.A., Noboru Okada M., Fumega U., Peliky Fontes M.A., Dutra Moraes M.F., Siqueira Haibara A. (2011). Excitatory amino acid receptors in the dorsomedial hypothalamus are involved in the cardiovascular and behavioural chemoreflex responses. Exp. Physiol..

[21] Morin S.M., Stotz-Potter E.H., DiMicco J.A. (2001). Injection of muscimol into dorsomedial hypothalamus and stress-induced Fos expression in paraventricular nucleus. Am. J. Physiol. Regul. Integr. Comp. Physiol..

[22] Briski K.P., Gillen E. (2001). Differential distribution of Fos expression within the male rat preoptic area and hypothalamus in response to physical vs. psychological stress. Brain Res. Bull..

[23] McDowall L.M., Horiuchi J., Killinger S., Dampney R.A. (2006). Modulation of the baroreceptor reflex by the dorsomedial hypothalamic nucleus and perifornical area. Am. J. Physiol. Regul. Integr. Comp. Physiol..

[24] Díaz-Casares A., López-González M.V., Dawid-Milner M.S., Baloyannis S.J., Gordeladze J.O. (2018). Role of the Dorso- and Ventrolateral Pons in Cardiorespiratory Hypothalamic Defense Responses. Hypothalamus in Health and Diseases.

[25] Silva N.T., Nalivaiko E., da Silva L.G., Haibara A.S. (2015). Excitatory amino acid receptors in the dorsomedial hypothalamic area contribute to the chemoreflex tachypneic response. Respir. Physiol. Neurobiol..

[26] Marins F.R., Limborço-Filho M., Xavier C.H., Biancardi V.C., Vaz G.C., Stern J.E., Oppenheimer S.M., Fontes M.A.P. (2016). Functional topography of cardiovascular regulation along the rostrocaudal axis of the rat posterior insular cortex. Clin. Exp. Pharmacol. Physiol..

[27] Oppenheimer S. (2007). Cortical control of the heart. Clevel. Clin. J. Med..

[28] Marins F.R., Iddings J.A., Fontes M.A.P., Filosa J.A. (2017). Evidence that remodeling of insular cortex neurovascular unit contributes to hypertension-related sympathoexcitation. Physiol. Rep..

[29] Oppenheimer S., Cechetto D. (2016). The insular cortex and the regulation of cardiac function. Compr. Physiol..

[30] Nagai M., Dote K., Kato M. (2021). Autonomic response after hemorrhagic stroke in the right insular cortex: What is the common pathophysiology in rat and human?. Auton. Neurosci..

[31] Marins F.R., Limborço-Filho M., D’Abreu B.F., Machado de Almeida P.W., Gavioli M., Xavier C.H., Oppenheimer S.M., Silvia Guatimosim S., Fontes M.A.P. (2020). Autonomic and cardiovascular consequences resulting from experimental hemorrhagic stroke in the left or right intermediate insular cortex in rats. Auton. Neurosci..

[32] Fontes M.A.P., Dos Santos Machado L.R., Viana A.C.R., Cruz M.H., Nogueira Í.S., Oliveira M.G.L., Neves C.B., Godoy A.C.V., Henderson L.A., Macefield V.G. (2024). The insular cortex, autonomic asymmetry and cardiovascular control: Looking at the right side of stroke. Clin. Auton. Res..

[33] Kataoka N., Shima Y., Nakajima K., Nakamura K. (2020). A central master driver of psychosocial stress responses in the rat. Science.

[34] LeDoux J. (2007). The amygdala. Curr. Biol..

[35] Soltis R.P., Cook J.C., Gregg A.E., Stratton J.M., Flickinger K.A. (1998). EAA receptors in the dorsomedial hypothalamic area mediate the cardiovascular response to activation of the amygdala. Am. J. Physiol..

[36] Zhang W., Zhang N., Sakurai T., Kuwaki T. (2009). Orexin neurons in the hypothalamus mediate cardiorespiratory responses induced by disinhibition of the amygdala and bed nucleus of the stria terminalis. Brain Res..

[37] Ressler K., Berretta S., Bolshakov V.Y., Rosso I.M., Meloni E.G., Rauch S.L., William A., Carlezon J.R. (2022). Post-traumatic stress disorder: Clinical and translational neuroscience from cells to circuits. Nat. Rev. Neurol..

[38] Bonnet L., Comte A., Tatu L., Millot J.L., Moulin T., Medeiros de Bustos E. (2015). The role of the amygdala in the perception of positive emotions: An “intensity detector”. Front. Behav. Neurosci..

[39] Thompson R.H., Swanson L.W. (1998). Organization of inputs to the dorsomedial nucleus of the hypothalamus: A reexamination with Fluorogold and PHAL in the rat. Brain Res. Brain Res. Rev..

[40] Horiuchi J., McDowall L.M., Dampney R.A. (2009). Vasomotor and respiratory responses evoked from the dorsolateral periaqueductal grey are mediated by the dorsomedial hypothalamus. J. Physiol..

[41] De Menezes R.C., Zaretsky D.V., Fontes M.A., DiMicco J.A. (2009). Cardiovascular and thermal responses evoked from the periaqueductal grey require neuronal activity in the hypothalamus. J. Physiol..

[42] Zafar T., Brouillard C., Lanfumey L., Sévoz-Couche C. (2018). A hypothalamo-midbrain-medullary pathway involved in the inhibition of the respiratory chemoreflex response induced by potassium cyanide in rodents. Neuropharmacology.

[43] Silva-Carvalho L., Dawid-Milner M.S., Goldsmith G.E., Spyer K.M. (1995). Hypothalamic modulation of the arterial chemoreceptor reflex in the anaesthetized cat: Role of the nucleus tractus solitarii. J. Physiol..

[44] Silva-Carvalho L., Dawid-Milner M.S., Spyer K.M. (1995). The pattern of excitatory inputs to the nucleus tractus solitarii evoked on stimulation in the hypothalamic defence area in the cat. J. Physiol..

[45] Silva-Carvalho L., Dawid-Milner M.S., Goldsmith G.E., Spyer K.M. (1993). Hypothalamic-evoked effects in cat nucleus tractus solitarius facilitating chemoreceptor reflexes. Exp. Physiol..

[46] Hosoya Y., Ito R., Kohno K. (1987). The topographical organization of neurons in the dorsal hypothalamic area that project to the spinal cord or to the nucleus raphe pallidus in the rat. Exp. Brain Res..

[47] Thompson R.H., Canteras N.S., Swanson L.W. (1996). Organization of projections from the dorsomedial nucleus of the hypothalamus: A PHA-L study in the rat. J. Comp. Neurol..

[48] Dampney R.A. (2015). Central mechanisms regulating coordinated cardiovascular and respiratory function during stress and arousal. Am. J. Physiol. Regul. Integr. Comp. Physiol..

[49] Linnman C., Moulton E.A., Barmettler G., Becerra L., Borsook D. (2012). Neuroimaging of the periaqueductal gray: State of the field. Neuroimage.

[50] Vaughn E., Eichhorn S., Jung W., Zhuang X., Dulac C. (2022). Three dimensional interrogation of cell types and instinctive behavior in the periaqueductal gray. bioRxiv.

[51] da Silva L.G., de Menezes R.C., dos Santos R.A., Campagnole-Santos M.J., Fontes M.A.P. (2003). Role of periaqueductal gray on the cardiovascular response evoked by disinhibition of the dorsomedial hypothalamus. Brain Res..

[52] de Menezes R.C., Zaretsky D.V., Fontes M.A., DiMicco J.A. (2006). Microinjection of muscimol into caudal periaqueductal gray lowers body temperature and attenuates increases in temperature and activity evoked from the dorsomedial hypothalamus. Brain Res..

[53] De Oliveira R.W., Del Bel E.A., Guimaraes F.S. (2000). Behavioral and c-fos expression changes induced by nitric oxide donors microinjected into the dorsal periaqueductal gray. Brain Res. Bull..

[54] Borelli K.G., Ferreira-Netto C., Brandao M.L. (2006). Distribution of Fos immunoreactivity in the rat brain after freezing or escape elicited by inhibition of glutamic acid decarboxylase or antagonism of GABA-A receptors in the inferior colliculus. Behav. Brain Res..

[55] Villela D.C., da Silva Junior L.G., Fontes M.A.P. (2009). Activation of 5-HT receptors in the periaqueductal gray attenuates the tachycardia evoked from dorsomedial hypothalamus. Auton. Neurosci..

[56] Johnson P.L., Lightman S.L., Lowry C.A. (2004). A functional subset of serotonergic neurons in the rat ventrolateral periaqueductal gray implicated in the inhibition of sympathoexcitation and panic. Ann. N. Y. Acad. Sci..

[57] Cao W.H., Fan W., Morrison S.F. (2004). Medullary pathways mediating specific sympathetic responses to activation of dorsomedial hypothalamus. Neuroscience.

[58] López-González M.V., González-García M., Peinado-Aragonés C.A., Barbancho M.A., Díaz-Casares A., Dawid-Milner M.S. (2020). Pontine A5 region modulation of the cardiorespiratory response evoked from the midbrain dorsolateral periaqueductal grey. J. Physiol. Biochem..

[59] González-García M., Carrillo-Franco L., Morales-Luque C., Dawid-Milner M.S., López-González M.V. (2024). Central Autonomic Mechanisms Involved in the Control of Laryngeal Activity and Vocalization. Biology.

[60] Hartmann K., Brecht M. (2020). A Functionally and Anatomically Bipartite Vocal Pattern Generator in the Rat Brain Stem. iScience.

[61] Subramanian H.H., Huang Z.G., Silburn P.A., Balnave R.J., Holstege G. (2018). The physiological motor patterns produced by neurons in the nucleus retroambiguus in the rat and their modulation by vagal, peripheral chemosensory, and nociceptive stimulation. J. Comp. Neurol..

[62] Fulwiler C.E., Saper C.B. (1984). Subnuclear organization of the efferent connections of the parabrachial nucleus in the rat. Brain Res..

[63] Dawid-Milner M.S., Lara J.P., Lopez de Miguel M.P., Lopez-Gonzalez M.V., Spyer K.M., Gonzalez-Baron S. (2003). A5 region modulation of the cardiorespiratory responses evoked from parabrachial cell bodies in the anaesthetised rat. Brain Res..

[64] Dutschmann M., Bautista T.G., Trevizan-Baú P., Dhingra R.R., Furuya W.I. (2021). The pontine Kölliker-Fuse nucleus gates facial, hypoglossal, and vagal upper airway related motor activity. Respir. Physiol. Neurobiol..

[65] Dutschmann M., Dick T.E. (2012). Pontine mechanisms of respiratory control. Compr. Physiol..

[66] Moga M.M., Herbert H., Hurley K.M., Yasui Y., Gray T.S., Saper C.B. (1990). Organization of cortical, basal forebrain, and hypothalamic afferents to the parabrachial nucleus in the rat. J. Comp. Neurol..

[67] Díaz-Casares A., López-González M.V., Peinado-Aragonés C.A., Lara J.P., González-Barón S.M., Dawid-Milner M.S. (2009). Role of the parabrachial complex in the cardiorespiratory response evoked from hypothalamic defense area stimulation in the anesthetized rat. Brain Res..

[68] Díaz-Casares A., López-González M.V., Peinado-Aragonés C.A., González-Barón S., Dawid-Milner M.S. (2012). Parabrachial complex glutamate receptors modulate the cardiorespiratory response evoked from hypothalamic defense area. Auton. Neurosci..

[69] Bruinstroop E., Cano G., Vanderhorst V.G., Cavalcante J.C., Wirth J., Sena-Esteves M., Saper C.B. (2012). Spinal projections of the A5, A6 (locus coeruleus), and A7 noradrenergic cell groups in rats. J. Comp. Neurol..

[70] López-González M.V., Díaz-Casares A., Peinado-Aragonés C.A., Lara J.P., Barbancho M.A., Dawid-Milner M.S. (2013). Neurons of the A5 region are required for the tachycardia evoked by electrical stimulation of the hypothalamic defence area in anaesthetized rats. Exp. Physiol..

[71] López-González M.V., Díaz-Casares A., González-García M., Peinado-Aragonés C.A., Barbancho M.A., Carrillo de Albornoz M., Dawid-Milner M.S. (2018). Glutamate receptors of the A5 region modulate cardiovascular responses evoked from the dorsomedial hypothalamic nucleus and perifornical area. J. Physiol. Biochem..

[72] Abbott S.B., Kanbar R., Bochorishvili G., Coates M.B., Stornetta R.L., Guyenet P.G. (2012). C1 neurons excite locus coeruleus and A5 noradrenergic neurons along with sympathetic outflow in rats. J. Physiol..

[73] Guyenet P.G. (2006). The sympathetic control of blood pressure. Nat. Rev. Neurosci..

[74] Dampney R.A.L., Coleman M.J., Fontes M.A.P., Hirooka Y., Horiuchi J., Li Y.W., Polson J.W., Potts P.D., Tagawa T. (2002). Central mechanisms underlying short-and longterm regulation of the cardiovascular system. Clin. Exp. Pharmacol. Physiol..

[75] Zhang H., Zhu Z., Ma W.X., Kong L.X., Yuan P.C., Bu L.F., Han J., Huang Z.L., Wang Y.Q. (2024). The contribution of periaqueductal gray in the regulation of physiological and pathological behaviors. Front. Neurosci..

[76] Li T.L., Chen J.Y.S., Huang S.C., Dai Y.W.E., Hwang L.L. (2018). Cardiovascular pressor effects of orexins in the dorsomedial hypothalamus. Eur. J. Pharmacol..

[77] Huang S.C., Dai Y.W., Lee Y.H., Chiou L.C., Hwang L.L. (2010). Orexins depolarize rostral ventrolateral medulla neurons and increase arterial pressure and heart rate in rats mainly via orexin 2 receptors. J. Pharmacol. Exp. Ther..

[78] Samuels B.C., Zaretsky D.V., DiMicco J.A. (2002). Tachycardia evoked by disinhibition of the dorsomedial hypothalamus in rats is mediated through medullary raphe. J. Physiol..

[79] Nogueira M.I., de Rezende B.D., do Vale L.E., Bittencourt J.C. (2000). Afferent connections of the caudal raphe pallidus nucleus in rats: A study using the fluorescent retrograde tracers fluorogold and true-blue. Anat. Anz..

[80] Krohn F., Novello M., van der Giessen R.S., De Zeeuw C.I., Pel J.J.M., Bosman L.W.J. (2023). The integrated brain network that controls respiration. eLife.

[81] Kanbar R., Orea V., Chapuis B., Barres C., Julien C. (2007). A transfer function method for the continuous assessment of baroreflex control of renal sympathetic nerve activity in rats. Am. J. Physiol. Regul. Integr. Comp. Physiol..

[82] Marina N., Turovsky E., Christie I.N., Hosford P.S., Hadjihambi A., Korsak A., Ang R., Mastitskaya S., Sheikhbahaei S., Theparambil S.M. (2018). Brain metabolic sensing and metabolic signaling at the level of an astrocyte. Glia.

[83] Amorim M.R., Wang X., Aung O., Bevans-Fonti S., Anokye-Danso F., Ribeiro C., Escobar J., Freire C., Pho H., Dergacheva O. (2023). Leptin signaling in the dorsomedial hypothalamus couples breathing and metabolism in obesity. Cell Rep..

[84] Dampney R.A., Furlong T.M., Horiuchi J., Iigaya K. (2013). Role of dorsolateral periaqueductal grey in the coordinated regulation of cardiovascular and respiratory function. Auton. Neurosci..

[85] Fontes M.A.P., Marins F.R., Patel T.A., de Paula C.A., Dos Santos Machado L.R., de Sousa Lima É.B., Ventris-Godoy A.C., Viana A.C.R., Linhares I.C.S., Xavier C.H. (2023). Neurogenic Background for Emotional Stress-Associated Hypertension. Curr. Hypertens. Rep..

[86] Esler M. (2017). Mental stress and human cardiovascular disease. Neurosci. Biobehav. Rev..

[87] Johnson H.M. (2019). Anxiety and Hypertension: Is There a Link? A Literature Review of the Comorbidity Relationship Between Anxiety and Hypertension. Curr. Hypertens. Rep..

[88] de Silva T., Cosentino G., Ganji S., Riera-Gonzalez A., Hsia D.S. (2020). Endocrine Causes of Hypertension. Curr. Hypertens. Rep..

[89] Altemus M., Redwine L.S., Leong Y.M., Frye C.A., Porges S.W., Carter C.S. (2001). Responses to laboratory psychosocial stress in postpartum women. Psychosom. Med..

[90] Korner P.I., Korner P.I. (2007). Psychosocial Stress and Hypertension. Essential Hypertension and Its Causes: Neural and Non-Neural Mechanisms.

[91] Sévoz-Couche C., Brouillard C., Camus F., Laude D., De Boer S.F., Becker C., Benoliel J.J. (2013). Involvement of the dorsomedial hypothalamus and the nucleus tractus solitarii in chronic cardiovascular changes associated with anxiety in rats. J. Physiol..

